# Effects of intensified training and tapering periods using different exercise modalities on judo-specific physical test performances

**DOI:** 10.5114/biolsport.2022.108702

**Published:** 2021-11-10

**Authors:** Ibrahim Ouergui, Imen Daira, Hamdi Chtourou, Anissa Bouassida, Ezdine Bouhlel, Emerson Franchini, Luca P. Ardigò

**Affiliations:** 1 High Institute of Sport and Physical Education, Kef, University of Jendouba, Tunisie; 2 Institut Supérieur du Sport et de l’Education Physique de Sfax, Université de Sfax, Tunisie; 3 Activité Physique, Sport et Santé, UR18JS01, Observatoire National du Sport, Tunis, Tunisie; 4 Laboratory of Cardio-Circulatory, Respiratory, Metabolic and Hormonal Adaptations to Muscular Exercise, Faculty of Medicine Ibn El Jazzar, University of Sousse, Sousse, Tunisia; 5 Martial Arts and Combat Sports Research Group, School of Physical Education and Sport, University of São Paulo, São Paulo, Brazil; 6 School of Exercise and Sport Science, Department of Neurosciences, Biomedicine and Movement Sciences, University of Verona, Verona, Italy

**Keywords:** Combat sports, Athletic assessment, Strength, Speed, High-intensity interval training

## Abstract

This study investigated the effects of intensified training and tapering periods using different exercise modalities on judo athletes’ physical fitness. Fifty-nine adolescent male and female judo athletes (age 15 ± 1 years) were randomly assigned to one of three experimental groups or one control group (CG). Experimental groups (kumi-kata [KG], uchi-komi [UG] and running [RG]) trained four times per week over four weeks of intensified training (in addition to their usual technical-tactical judo training; CG underwent only the usual training) followed by 12 days of tapering. The countermovement jump test (CMJ), isometric and dynamic judogi chin-up tests (JCT), uchi-komi speed test (UST), Special Judo Fitness Test (SJFT) and Judo Physical Fitness Test (JPFT) were administered before and after the intensified period and after tapering. The CMJ performance was superior in KG compared with UG, RG and CG. Isometric JCT performance was superior in KG compared with RG and CG. Regarding UST, performance was superior in UG compared with RG and CG. The same performance was superior with respect to the same groups considering pre-training to tapering change. The SJFT index did not differ between groups or time-points. The JPFT index increased after intensified and tapering periods compared with before training, with KG eliciting higher values compared with CG. Coaches and conditioning coaches could administer kumi-kata high-intensity interval training to enhance athletes’ judo-specific physical fitness.

## INTRODUCTION

To overcome physiological and physical loads and allow technical-tactical success during judo competitions, which are distributed in an elevated number throughout the annual season, coaches need to develop physical abilities (i.e., power, strength and endurance) during intensified training periods followed by recovery and tapering periods [1]. Therefore, coaches and physical conditioning coaches try to improve their training programmes by adding stimuli in terms of training methods that may allow judo athletes to achieve positive adaptation. However, most of the studies about high intensity interval training (HIIT) in judo athletes used running as the exercise mode [2–4], as high-level judo athletes frequently engage in this kind of exercise [5]. However, training specificity has been considered extremely important for judo-specific performance improvement [6]. Specificity can still be guaranteed by administrating training exercises complying with the time structure of judo sport [7] and/or by administrating specific exercises engaging athletes in specific judo movements (e.g., combat simulation [randori], kumi-kata [i.e., grip dispute] and technical preparation [uchi-komi and nage-komi, i.e., repeated throwing techniques]) and concomitant physical conditioning sessions [5, 8]. Taking into consideration the above-mentioned information, HIIT was reported to be among the most suitable conditioning modalities usable for judo training sessions especially when specific movements (i.e., uchi-komi) were integrated [6]. Specifically, it has been shown that short-term (4-week) and low-volume (two blocks of 10 × 20 s/10 s all-out exercise) HIIT protocols (i.e., lower-body, upper-body cycle ergometer exercises and uchi-komi) added to regular judo training (i.e., habitual technical-tactical judo training) improve performance in the Special Judo Fitness Test (a test used to evaluate judo-specific fitness using throwing techniques; SJFT [6]) and aerobic and anaerobic performances especially in the uchi-komi group, the only group improving both lower and upper-body aerobic and anaerobic performances [9].

To the authors’ current knowledge, there is no study that has investigated and compared the effects of HIIT between different judo-specific training modalities (e.g., uchi-komi vs. kumi-kata) and with generic exercises regarding different judo performance aspects followed by a tapering period. Thus, the aim of the present study was to investigate the effects on specific judo performances of four weeks of HIIT added to regular judo training followed by 12 days of tapering using different exercise modalities (namely uchi-komi, kumikata and running). It was hypothesized that kumi-kata training would result in better isometric and dynamic strength-endurance judogi chin-up tests as well as the Special Judo Fitness Test and the Judo Physical Fitness Test, whereas uchi-komi training would induce better uchi-komi test performance. Based on the training specificity principle, it was hypothesized that both kumi-kata and uchi-komi would induce greater improvement in comparison with the running modality as most of the tests used in the present study were judo-specific.

## MATERIALS AND METHODS

Using G*Power software (Heinrich-Heine-Universität, Düsseldorf, Germany) with α and power fixed at 0.05 and 0.95, respectively, and effect size fixed at 0.3 based on the study of Franchini et al. [9], sample size was calculated a priori. The required sample size was 40, but a higher number of participants was recruited considering the possibility of dropouts during the training process. Fifty-nine male (n = 35) and female (n = 24) judo athletes volunteered to participate in the study. Athletes were randomly assigned to kumikata (n = 14; mean ± standard deviation; age 16 ± 1 years, height 171.0 ± 0.8 cm and body mass 58.4 ± 7.4 kg), uchikomi (n = 16, age 15 ± 1 years, height 164.2 ± 0.8 cm and body mass 58.9 ± 8.3 kg), running (n = 17, age 15 ± 1 years, height 162.5 ± 1.1 cm and body mass 55.8 ± 3.2 kg) and a control group (n = 12, age 15 ± 0.5 years, height 159.2 ± 0.5 cm and body mass 56.8 ± 5.4 kg). Athletes were training four times per week (1 h 30 min/session), had more than seven years of experience in judo practice and were participating regularly in national judo championships. They did not present any medical restrictions during the whole period of the experimentation. After receiving a thorough explanation of the risks and benefits of the study, athletes and their parents gave written informed consent and the study was conducted according to the Declaration of Helsinki for Human Experimentation [10] and the protocol was fully approved by the local research ethics committee.

The study aimed at comparing the effects of three HIIT modalities (i.e., kumi-kata, uchi-komi and running) during intensified training and tapering periods on physical and physiological characteristics in judo athletes. This study shared some procedures and most of the participants with a previous investigation aimed at assessing the effects of intense and tapering training periods using different judo-specific and general exercise modalities on rating of perceived exertion, well-being indices, recovery state and physical enjoyment [17]. Athletes were submitted to 10 days of rest to avoid any potential effect of a previous training programme on judo athletes’ performance. After that, before the beginning of the experimentation, athletes were familiarized with testing procedures and all sessions were performed at the same time of day to avoid diurnal variation of the performance [11]. Specific judo performance was assessed using the Special Judo Fitness Test (SJFT [12]) and the Judo Physical Fitness Test (JPFT; see description below [13]). Briefly, the SJFT consists of three periods (A = 15 s, B and C = 30 s) with 10 s intervals between them. During each period the athlete (tori) under evaluation throws two partners (uke A and B, 6 m from each other) as many times as possible using the ippon-seoi-nage technique (a hand throwing technique). The Judo Physical Fitness Test was specifically designed to allow assessment of all the main fundamental actions of judo (i.e., grips, throws and chokes) executed at high intensity and intermittently, similarly to the SJFT [13]. Moreover, upper-body strength-endurance (with isometric and dynamic judogi chin-up tests [14]), judo uchi-komi-specific speed [15] and jumping height (with countermovement jump test [CMJ test] [16]) were assessed. After the baseline assessment, athletes were randomly assigned to three experimental groups (i.e., kumi-kata, uchi-komi and running) and a control group (habitual technical-tactical judo training). Kumi-kata (i.e., grip dispute in the standing combat phase with no throwing technique execution), uchi-komi (i.e., performing repetitive technical entrance without throwing with a single partner belonging to the same weight category) and running (i.e., running back and forth as fast as possible between two lines separated by 6 m [17]) groups performed four weeks of intensified training consisting of additional HIIT sessions in addition to the regular training programme. During such sessions, athletes performed two blocks of 10 sets of 20 s allout efforts interspersed by 10 s intervals between sets and 5 min between blocks [9]. After the intensified training period, athletes were submitted to a 12-day tapering training period with the training volume being reduced by half including HIIT sessions during which only one block was performed by athletes. Post-assessment sessions for all groups were performed 72 h after the final training session to ensure sufficient recovery for athletes. All tests were performed during different sessions (days) allowing sufficient recovery periods (24 h) to avoid any fatigue effects between sessions. Countermovement jump test, uchi-komi speed test (UST), and isometric and dynamic strength-endurance chin-up tests were performed during the first session, whereas SJFT and JPFT were performed on the second testing day. A sufficient recovery period was given to all athletes (≥ 30 min) when different tests were performed on the same day. Internal training load, well-being and recovery for each training protocol were monitored and these outcomes were reported in previous publication using a slightly bigger sample size (n = 61; i.e., with two additional athletes [17]).

### Countermovement jump test

Regarding the CMJ test, from a standing position, athletes completed a fast downward movement by flexing the knees and hips, followed by rapid extension of both knees and hips. Height jump performance was recorded over three trials using an Optojump (Optojump, Microgate, Bolzano, Italy), with 45 s of passive recovery between jumps, and the best performance was kept for further analysis. The test was repeated for test-retest precision assessment. The intraclass correlation coefficient value was 0.933. The typical error of measurement was 1.7 cm.

### Isometric and dynamic strength-endurance judogi chin-up tests

Regarding the isometric strength-endurance judogi chin-up test, athletes were asked to keep their elbows flexed with the chin above the hands for as long as possible and the holding time was registered with 0.1 s accuracy. The test was interrupted as soon as athletes became unable to maintain the initial holding isometric position. Regarding the test’s dynamic version, judo athletes had to fully extend their elbows and then flex them until the chin was above the position of the hands with completely correct repetitions being counted. The test was stopped when athletes were no longer able to perform correct repetitions or stopped voluntarily. Tests were repeated for test-retest precision assessment. The intraclass correlation coefficient values regarding isometric and dynamic strength-endurance judogi chin-up tests were 0.80 and 0.76, respectively. Typical error of measurement values were 2.3 s and 0.3 repetitions, respectively.

### Uchi-komi speed test

Regarding the uchi-komi speed test, athletes were instructed to repeat as fast and as many times as possible the ippon-seoi-nage technique without throwing over 15 s [15]. The test was repeated for test-retest precision assessment. The intraclass correlation coefficient value was 0.88. Typical error of measurement was 1 repetition.

### Special Judo Fitness Test

As stated above, the SIFT was divided into three periods (A = 15 s and B and C = 30 s) with rest intervals of 10 s in between. During each period, the evaluated athlete had to run towards two partners 6 m away from each other and throw each athlete as many times as possible using the ippon-seoi-nage technique. All athletes had to have similar height and body mass. Heart rate was measured just after the test (HR1) and one minute later (HR2). Throws were counted and the SJFT index was calculated for Equation 1 and used for further analysis. The test was repeated for test-retest precision assessment. The intraclass correlation coefficient value was 0.82 for throws and 0.87 for the SJFT index. Typical error of measurement values were 0.7 for throws and 0.4 beats·min^-1^·throws^-1^ for the index.


$$\text{SJFT index }\left[\text{beats}\cdot {\text{min}}^{-1}\cdot {\text{throws}}^{-1}\right]=\frac{\text{HR}1\left[\text{beats}\cdot {\text{min}}^{-1}\right]+\text{HR}2\left[\text{beats}\cdot {\text{min}}^{-1}\right]}{\text{Nb throws}}$$ (Equation 1)

### Judo Physical Fitness Test

Regarding JPFT, the test procedure needed a judogi (i.e., traditional judo uniform) hanging from a high bar and three judo athletes to act as uke (i.e., those receiving the techniques) belonging to the same weight category as tori (i.e., the athlete being evaluated). Four markers were placed at a distance of 1.5 m from the centre of the test area. Two uke were placed opposite each other (at a distance of 3 m from each other), whereas the third one lay on the chest opposite to the judogi hanging from the bar (at a distance of 3 m, as well). The test was divided into three periods lasting 30 s of work for each one with 10 s rest intervals in between. Each set was composed of 10 s of isometric effort (i.e., the judo athlete grips with extended elbows the reverse of judogi) and 20 s of executing as many times as possible a throwing technique (i.e., ippon-seoi-nage) and the yoko-sankaku-jime (i.e., a choking technique). The numbers of executions of each series were counted and their total number (n) was used for index calculation. Heart rate was measured immediately after the test (HR1) and one minute later (HR2). The test was repeated for test-retest precision assessment. The intraclass correlation coefficient value was 0.80. Typical error of measurement was 0.5 beats·min^-1^·execution^-1^. The Judo Physical Fitness Test was calculated as follows (Equation 2):

$$\text{JPFT index }\left[\text{beats}\cdot {\text{min}}^{-1}\cdot {\text{execution}}^{-1}\right]=\frac{\text{HR}1\left[\text{beats}\cdot {\text{min}}^{-1}\right]+\text{HR}2\left[\text{beats}\cdot {\text{min}}^{-1}\right]}{\text{Nb execution}}$$ (Equation 2)

### Statistical analyses

Data were presented as mean and standard deviation. The statistical analysis was performed using SPSS 20.0 statistical software (IBM, Armonk, USA). The normality of data sets was checked and confirmed using the Kolmogorov-Smirnov test. Sphericity was tested and confirmed using the Mauchly test. Performances at pre-training were compared between groups by means of a one-way analysis of variance. Regarding variables (CMJ, isometric and dynamic strength-endurance judogi chin-up tests and number of throws and JPFT HR1 and HR2) that did not differ between groups at pre-training, data were analysed using a two-way analysis of variance (group [kumikata, uchi-komi, running and control group] × training [before and after intensified period and after tapering]) with repeated measurements to compare test performances. The Bonferroni test was used as a post-hoc test. Regarding variables that differed between groups at pre-training (uchi-komi speed test, number of throws, SJFT HR1and HR2), delta values between pre-training and after the intensified training period and between pre-training and after tapering were calculated and these values were compared by means of a two-way (factor 1: 4 groups and factor 2: 2 time-points) analysis of variance with repeated measurements in the second factor followed by the Bonferroni test. Moreover, the delta value between pre-training and after tapering was also calculated and compared through a one-way analysis of variance followed by the Tukey test for unequal n. The magnitude of differences between variables was interpreted using the standardized effect size (Cohen’s d) classified according to Hopkins [18] as follows: d ≤ 0.20 (trivial), 0.20 < d ≤ 0.60 (small), 0.60 < d ≤ 1.20 (moderate), 1.20 < d ≤ 2.0 (large), 2.0 < d ≤ 4.0 (very large) and d > 4.0 (extremely large). Furthermore, upper and lower 95% confidence intervals of difference (95%CI_d_s) were calculated for corresponding variations. The statistical significance level was set at p ≤ 0.05.

## RESULTS

The groups did not differ significantly in terms of age, height or body mass (p > 0.05 regarding all comparisons). Table 1 presents CMJ, isometric and dynamic judogi chin-up test performance values recorded before and after intensified training and after tapering. Regarding CMJ, a group effect was found (F_3,165_ = 12.42; p < 0.001) with the kumi-kata group presenting higher values (p < 0.001 for all comparisons) compared with uchi-komi (95%CI_d_ = 1.4,6.7; d = 0.73, moderate), running (95%CI_d_ = 2.1,7.4; d = 0.91, moderate) and control groups (95%CI_d_ = 3.1,8.8; d = 1.2, moderate).

**TABLE 1:** Countermovement jump (CMJ), isometric and dynamic strength-endurance judogi chin-up tests performances after intensified training and tapering periods in kumi-kata, uchi-komi, running and control groups (values are presented as mean ± standard deviation).

	Kumi-kata group (n = 14)			Uchi-komi group (n = 16)			Running group (n = 19)			Control group (n = 12)		
	Pre-IT	Post-IT	Post-Tap	Pre-IT	Post-IT	Post-Tap	Pre-IT	Post-IT	Post-Tap	Pre-IT	Post-IT	Post-Tap
**CMJ (cm)**	31.0 ± 5.7	35.0 ± 6.2*	35.3 ± 7.0*	29.6 ± 3.7	29.6 ± 5.0	30.0 ± 5.4	29.1 ± 3.6	29.8 ± 4.1	28.2 ± 4.5	28.9 ± 1.4	27.3 ± 3.0	27.1 ± 3.4
**Isometric judogi chin-up test (s)**	57.8 ± 24.1^†,¶^	60.2 ± 22.4^†,¶^	56.6 ± 10.5^†,¶^	56.6 ± 10.5	53.8 ± 12.1	53.7 ± 12.7	48.4 ± 4.3	49.3 ± 4.1	50.1 ± 4.4	49.3 ± 13	49.8 ± 12.3	50.2 ± 11.8
**Dynamic judogi chin-up test (rep)**	25 ± 9	28 ± 11	25 ± 3	25 ± 3	25 ± 3	23 ± 5	26 ± 5	25 ± 4	26 ± 4	27 ± 8	26 ± 7	26 ± 7

* main effect of group, kumi-kata differed from other groups (p < 0.001);

† main effect of group, kumi-kata differed from running group (p = 0.013);

¶ main effect of group, kumi-kata differed from control group (p = 0.045). IT: intensified training; TAP: tapering; rep: repetition.

Regarding the isometric strength-endurance judogi chin-up test, a group effect was detected (F_3,165_ = 4.15; p = 0.007) with higher values for the kumi-kata group in comparison with running (95%CI_d_ = 1.3,6.8; d = 0.60, small; p = 0.013) and control groups (95%CI_d_ = 0.1,17.0; d = 0.48, small; p = 0.045). Moreover, no time-point (F_2,165_ = 0.30; p = 0.745), no group (F_3,165_ = 1.23; p = 0.300) and no interaction effect were found (F_6,156_ = 0.35; p = 0.910) regarding the dynamic strength-endurance judogi chin-up test.

Table 2 presents performance values recorded in UST, SJFT and the JPFT before and after the intensified training period and after tapering. Regarding UST, the delta values differed between groups (F_3,55_ = 7.59; p < 0.001) with higher delta values for the uchikomi group compared with running (95%CI_d_ = 1.7,2.5; d = 1.28, large; p = 0.001) and control groups (95%CI_d_ = 1.6,2.7; d = 1.49, large; p < 0.001). Additionally, when pre-training-to-tapering change was calculated, a significant difference was found between groups (F_3,55_ = 7.59; p < 0.001) with larger increments for the uchi-komi group compared with running (95%CI_d_ = 2.1,2.3; d = 0.76, moderate; p = 0.002) and control groups (95%CI_d_ = 2.1,2.7; d = 0.95, moderate; p = 0.002).

**TABLE 2:** Uchi-komi speed test (UST), Special Judo Fitness Test (SJFT) and Judo Physical Fitness Test (JPFT) performances after intensified training and tapering periods in kumi-kata, uchi-komi, running and control groups (values are presented as mean ± standard deviation).

		Kumi-kata group (n = 14)			Uchi-komi group (n = 16)			Running group (n = 17)			Control group (n = 12)		
		Pre-IT	Post-IT	Post-Tap	Pre-IT	Post-IT	Post-Tap	Pre-IT	Post-IT	Post-Tap	Pre-IT	Post-IT	Post-Tap
	**UST (rep)**	16 ± 2	18 ± 2	18 ± 1	15 ± 1	17 ± 1*,^†^	18 ± 1*,^†,¶^	15 ± 1	15 ± 1	16 ± 1	14 ± 2	14 ± 2	15 ± 1

**SJFT**	Throws (rep)	25 ± 4	26 ± 2	28 ± 3	23 ± 2	25 ± 2	26 ± 2	22 ± 2	24 ± 2	26 ± 2	20 ± 1	22 ± 1	23 ± 1
	HR1 (beats·min^-1^)	188 ± 5	186 ± 4	179 ± 5	195 ± 8	191 ± 5	185 ± 7	195 ± 10	189 ± 8	187 ± 8	190 ± 6	189 ± 4	187 ± 4
	HR2 (beats·min^-1^)	164 ± 11	154 ± 9	144 ± 12	171 ± 11	164 ± 14	154 ± 16	168 ± 15	156 ± 12	153 ± 13	164 ± 23	157 ± 12	152 ± 9
	Index (beats·mm^-1^·throws^-1^)	14.5 ± 2.5	13.1 ± 1.3	11.9 ± 1.5	16.0 ± 1.4	14.4 ± 1.4	13.3 ± 1.3	16.7 ± 2.2	14.5 ± 1.6	13.3 ± 1.5	17.4 ± 1.8	15.6 ± 0.8	14.6 ± 0.9

**JPFT**	Throws (rep)	23 ± 3^b^	26 ± 3^a,b^	29 ± 2^a,b,f^	22 ± 3^b,c^	25 ± 3^a,c^	27 ± 4^a,c^	20 ± 2	24 ± 2^a^	26 ± 2	20 ± 1	22 ± 2^a^	24 ± 1^a^
	HR1 (beats^-1^·min^-1^)	185 ± 5^f^	181 ± 4^f^	177 ± 4^d,e,f^	193 ± 5	189 ± 7	184 ± 6^d,e^	192 ± 5	188 ± 7	184 ± 6^d,e^	193 ± 5	190 ± 6	187 ± 5^d,e^
	HR2 (beats^-1^·min^-1^)	163 ± 8^i^	145 ± 16^h,i^	143 ± 12^a,g,i^	163 ± 12	155 ± 14^h^	147 ± 8^a,g^	164 ± 14	157 ± 13^h^	148 ± 11^a,g^	165 ± 12	163 ± 7^h^	152 ± 12^a,g^
	Index (beats^-1^·min^-1^·execution^-1^)	2.9 ± 1.1^j^	5.1 ± 2.2^e,j^	5.7 ± 1.9^a,j^	3.3 ± 1.5	5.7 ± 4.8^e^	5.4 ± 1.4 ^a^	2.9 ± 1.5	4.0 ± 1.8^e^	5.0 ± 1.6^a^	3.0 ± 1.2	3.1 ± 0.9^e^	4.6 ± 1.7^a^

* main effect of group: uchi-komi differed from running group (p = 0.001);

† main effect of group: uchi-komi differed from control group (p < 0.001);

¶ main effect of group: uchi-komi differed from running and control groups for delta values between pre-training and tapering (p = 0.002);

a main effect of time-point with post-Tap differing from pre-IT and post-IT and post-IT differing from pre-IT (p < 0.001);

b main effect of group with kumi-kata differing from running and control groups (p < 0.001);

c main effect of group with uchi-komi differing from control group (p = 0.002);

d main time-point effect with values post-Tap differing from post-IT (p = 0.001);

e main time-point effect with values differing from pre-IT (p = 0.001);

f main group effect with kumi-kata differing from all other groups (p < 0.001);

g main time-point effect with values post-Tap differing from post-IT (p = 0.002);

h main time-point effect with values post-IT differing from pre-IT (p = 0.01);

i main group effect with kumi-kata differing from control group (p = 0.015);

j main group effect with kumi-kata differing from control group (p = 0.046).

IT: intensified training; TAP: tapering; rep: repetition; HR1: heart rate immediately after the test; HR2: heart rate 1 min after the test.

Regarding SJFT, changes (pre-training-to-intensified training and intensified training-to-tapering) for number of throws, HR1, HR2 and index did not differ between groups or time-points and no group by time-point interaction were found (p > 0.101). Additionally, when pre-training-tapering change was calculated, no significant differences were found between groups regarding the same variables (p > 0.322).

Regarding JPFT, the number of throws differed across time (F_2,165_ = 47.85; p < 0.001) with increased values after tapering in comparison with after intensified training (95%CI_d_ = 1,3; d = 0.72, moderate; p < 0.001) and before the training period (95%CI_d_ = 4,6; d = 1.69, large; p < 0.001) and increased values after the intensified training period in comparison with before training (95%CI_d_ = 1,4; d = 0.98, moderate; p < 0.001). Moreover, the number of throws differed between groups (F_3,165_ = 12.92; p < 0.001) with the kumikata group eliciting higher values than running (95%CI_d_ = 1,4; d = 0.72, moderate; p < 0.001) and control groups (95%CI_d_ = 2,5; d = 1.28, large; p < 0.001) and the uchi-komi group showing higher values than the control group (95%CI_d_ = 1,4; d = 0.65; moderate; p = 0.002). Regarding HR1, there was a time-point effect (F_2,165_ = 14.14; p < 0.001) with decreased values after tapering in comparison with after intensified training (95%CI_d_ = -9,-3; d = 0.9; moderate; p < 0.001) and before training periods (95%CI_d_ = -7,-1; d = 0.6, moderate; p = 0.001). Moreover, a group effect was found (F_3,165_ = 13.30; p < 0.001) with the kumi-kata group showing lower values than running (95%CI_d_ = -9,-2; d = 0.87, moderate; p < 0.001), uchi-komi (95%CI_d_ = -10,-3; d = 1.0, moderate; p < 0.001) and control groups (95%CI_d_ = -11,-4; d = 1.4, large; p < 0.001). Regarding HR2, there was a time-point effect (F_2,165_ = 26.37; p < 0.001) with decreased values after tapering in comparison with after intensified training (95%CI_d_ = -13,-2; d = 0.6, moderate; p = 0.002) and before training periods (95%CI_d_ = -22,-11; d = 1.4, large; p < 0.001) and decreased values after the intensified period in comparison to before training (95%CI_d_ = -14,-3; d = 0.65; moderate; p = 0.01). Moreover, a group effect was found (F_3,165_ = 4.69; p = 0.04) with the kumikata group showing lower values than the control group (95%CI_d_ = -17,-3; d = 0.7, moderate; p = 0.015). Finally, regarding JPFT index, a time-point effect was detected (F_2,165_ = 15.18; p < 0.001) with lower values before training compared with after intensified training (95%CI_d_ = 0.5,2.4; d = 0.6, moderate; p = 0.001) and tapering periods (95%CI_d_ = 1.2,3.1; d = 1.4; large; p < 0.001). Likewise, a group effect was detected (F_3,165_ = 2.86; p = 0.037) with the kumi-kata group showing higher values in comparison to the control group (95%CI_d_ = 0.01,2.0; d = 0.53, small; p = 0.046).

## DISCUSSION

The present study showed that CMJ performance in the kumi-kata group was superior compared to uchi-komi, running and control groups and that isometric judogi chin-up test performance in kumikata was superior compared to running and control groups. Moreover, uchi-komi speed test improvement (i.e., delta between pre-training and post-training and delta between pre-training and post-tapering) was greater in the uchi-komi group in comparison with running and control groups. The Special Judo Fitness Test index did not differ between groups or time-points. Moreover, the JPFT index increased after intensified training and tapering periods in comparison with before training, with kumi-kata eliciting higher values in comparison to the control group.

In the present study, CMJ performance did not improve after the two periods of training. Similarly, Papacosta et al. [19] reported that jumping performance did not improve after 2 weeks of intensive judo training. However, improvements were observed after two weeks of tapering. Similarly, Ouergui et al. [20] found that four weeks of either repeated sprint training or repeated high-intensity technique training in young taekwondo athletes did not improve CMJ performance. By contrast, Monks et al. [21] found that 11 sessions of running HIIT performed over four weeks in taekwondo athletes elicited improvements in jumping performance. In fact, HIIT was reported to induce generally only trivial changes in muscle power [22, 23]. It was also reported that HIIT training volume, frequency, the lack of overload and specific training aiming at developing muscle power are among the factors limiting desired improvement [24]. Consequently, technical aspects of jump performance may need to be incorporated within HIIT if meaningful improvements in power performance assessed via CMJ are pursued [24]. On the other hand, the kumi-kata group showed higher values compared to the other training groups. However, this was detected only via a main effect of group and cannot be attributed to any specific adaptations in response to this training mode.

Regarding isometric and dynamic strength-endurance chin-up tests, the present study showed that performances did not differ after the training periods. Similarly, it was reported that grip isometric maximal strength of the right hand did not change across intensive and tapering judo training periods. Yet, the same study showed that the grip strength of the left hand increased after both intensified training and tapering periods [19]. In the present study, the kumikata group elicited superior performance in the isometric judogi chin-up test compared with running and control groups. However, again, this was only a main effect of group and this difference cannot be attributed to any specific adaptation in response to the specific kumikata training mode.

Regarding the uchi-komi speed test, the present study found that the delta values across different times of training did not vary, showing that the improvements were similar over the two training phases (i.e., intensified training and tapering). Indeed, the uchi-komi group achieved greater improvement in comparison with running and control groups. This may be the result of the techniques’ repetition with speed and power over uchi-komi performed by this group resulting in a higher number of repetitions performed per time. This shows that the specificity training principle was confirmed via this test.

The Special Judo Fitness Test is the most used test to specifically evaluate judo athletes [25]. Within the present study, the SJFT index did not differ between groups or time-points. A similar result was obtained in the study of Papacosta et al. [19], which showed that the SJFT index did not improve after intensified judo training or tapering periods. However, it was reported that HIIT added to regular judo training contributed to enhanced specific performance of judo athletes assessed by the SJFT [6]. Therefore, more studies are needed to verify which factors effectively affect the improvement in the SJFT after some short-term training.

Finally, this is the first study to assess judo athletes’ fitness using the JPFT. The JPFT index increased after both intensified training and tapering periods. This may be the result of both aerobic and anaerobic fitness improvements, as they both contribute to performance during this test, as reported regarding SJFT in a previous study [6], and it is acknowledged that JPFT and SJFT are highly correlated with each other [13]. The JPFT index improvement after intensified training and tapering periods reflects the physiological benefits of these two periods witnessed by the increase in the number of throws as well as in the decrease in HR values. Moreover, as reported regarding SJFT, the kumi-kata group elicited a test index higher than the control group, but this was a main effect of group, and it is difficult to attribute such an adaptation to this specific training mode.

Finally, we acknowledge a couple of limitations of the current study. In fact, while this study investigated the effects of these judo-specific HIIT protocols on the performance aspects of judo, specific competition performance indicators (e.g., number of applied techniques and time-motion variables) as well as adaptation of physiological responses were not taken into account in terms of training outcome. Another study limitation was that maturational control was not considered.

## CONCLUSIONS

The present study showed that technique repetition performance and the JPFT index improved after both intensified training and tapering periods. The kumi-kata group elicited better performance in the CMJ test, isometric judogi chin-up test and JPFT, but this was only a main effect of group. Nevertheless, the uchi-komi group elicited better performance in the uchi-komi speed test compared to running and control groups. These results are in agreement with the findings from a recent review [26] and support the effectiveness of short-term HIIT added to a regular judo training regime in improving athletes’ judo-specific performance, whereas the uchi-komi modality is more effective to improve specific speed compared with running and regular training regimes. Thus, coaches and strength and conditioning professionals could use these sport-specific HIIT training protocols when aerobic and anaerobic improvements are pursued. Furthermore, HIIT cannot be an effective training modality aiming at specifically developing strength and power, which can be improved more effectively using other exercises.
